# Supplementary material for: Long non-coding RNA DARS-AS1 promotes tumor progression by directly suppressing PACT-mediated cellular stress
Source: Commun Biol. 2022 Aug 15;5:822. doi: 10.1038/s42003-022-03778-y (PMC9378715; doi:10.1038/s42003-022-03778-y)
Supplement: Supplementary file 2 — Supplementary Information [file 42003_2022_3778_MOESM2_ESM.pdf]

Supplementary Figure 1

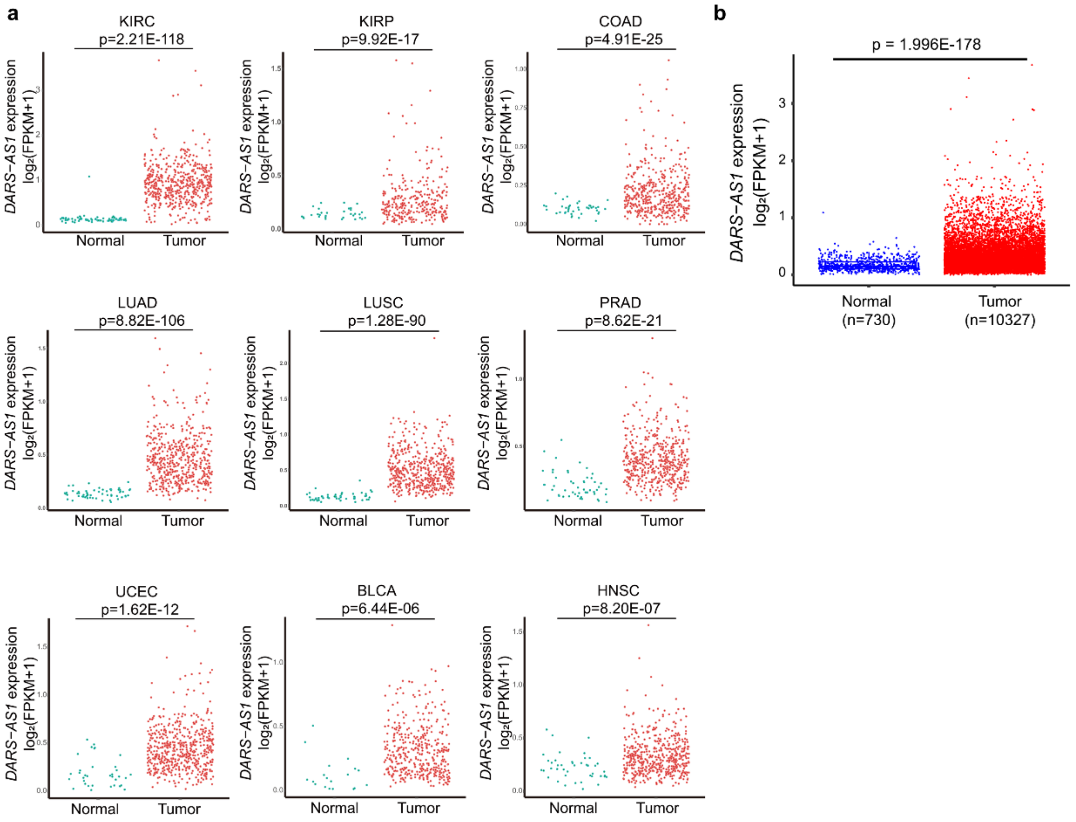

Supplementary Fig. 1. *DARS-AS1* is up-regulated in tumor samples. a, Expression of *DARS-AS1* in normal and tumor samples from KIRC, KIRP, COAD, LUAD, LUSC, PRAD, UCEC, BLCA and HNSC patients, respectively. **b**, Expression of *DARS-AS1* in all cancerous (n=10,327) and healthy samples (n=730). P-values were obtained by unpaired two-tailed Student's *t* test (from TCGA database).

Supplementary Figure 2

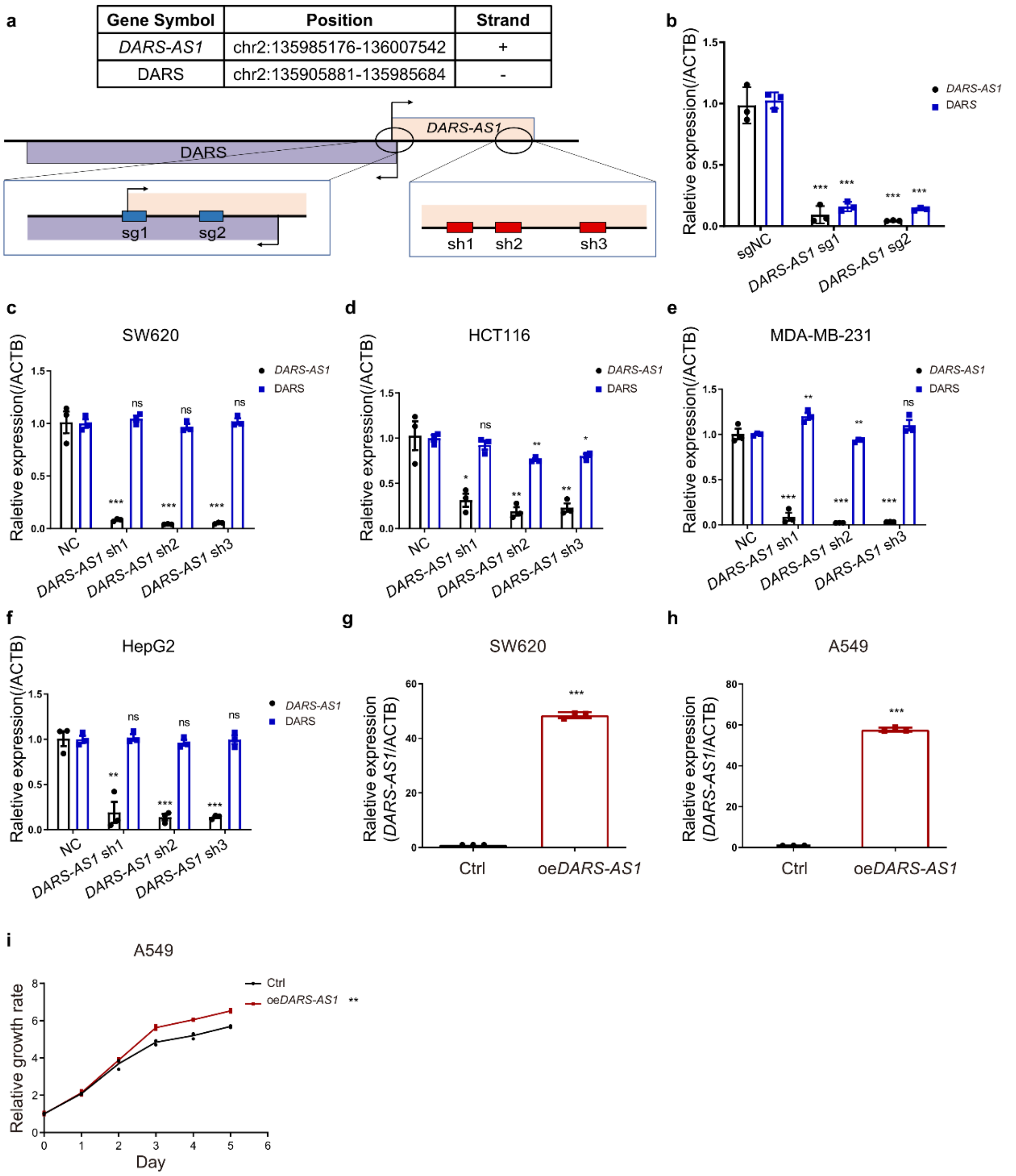

Supplementary Fig. 2. Expression of *DARS-AS1* effects cell proliferation of multiple cancers. a, Schematic representation of *DARS-AS1* and *DARS* genomic locus. **b,** Validation of knockdown efficiency of *DARS-AS1* and *DARS* by sgRNAs in SW620. **c-f**, Knockdown efficiency of *DARS-AS1* and DARS in SW620, HCT116, MBA-MD-231, and HepG2 cells by shRNAs. **g-h**, RT-qPCR validation for overexpression of *DARS-AS1* in SW620 and A549 cells. **i**, Overexpression of *DARS-AS1* promotes A549 cell proliferation. Data shown are means ± SD in triplicates experiments. *p ≤ 0.05, **p ≤ 0.01, ***p ≤ 0.001, **** p < 0.0001, ns, not significant, by two-tailed Student's *t test*.

Supplementary Figure 3

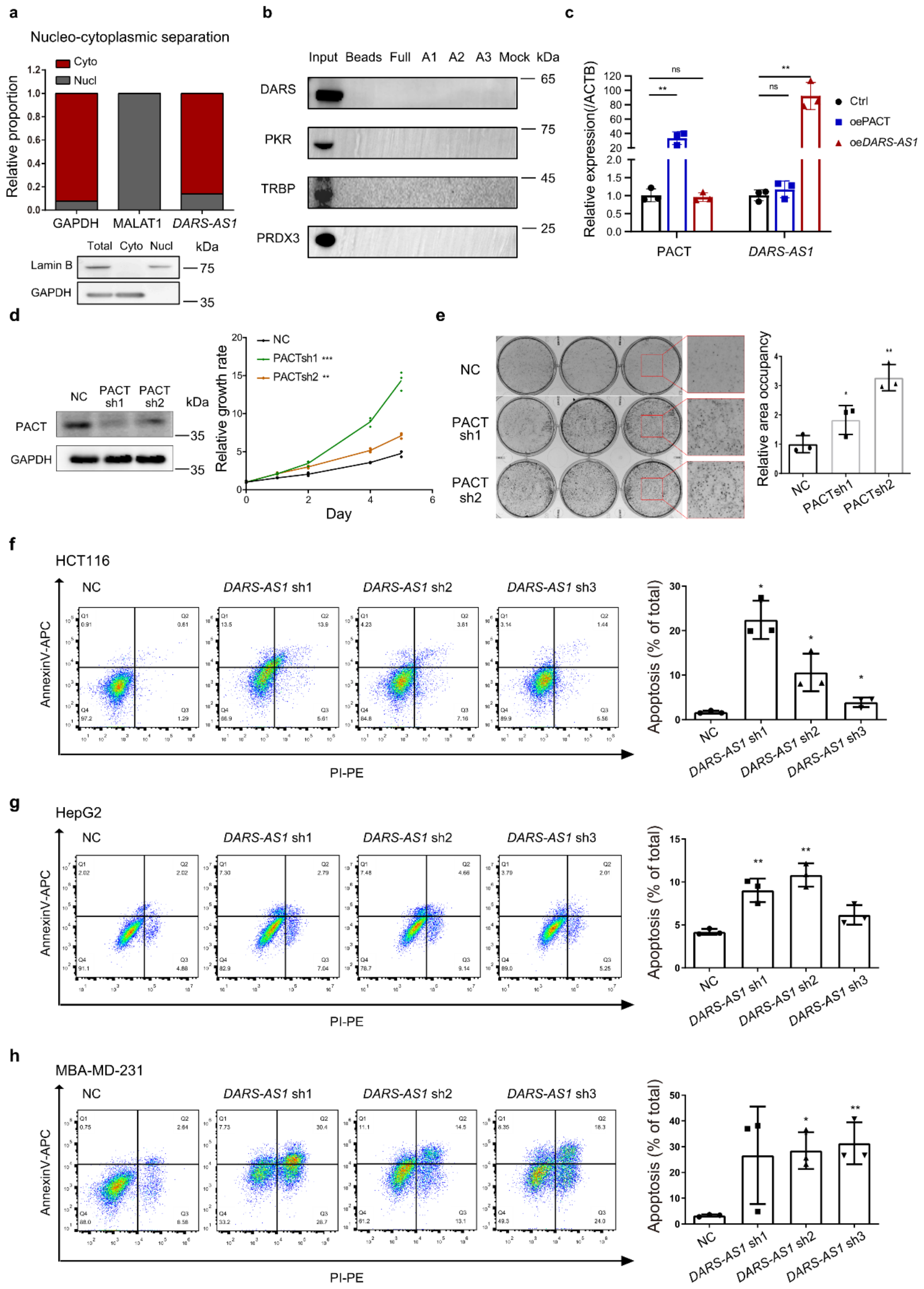

Supplementary Fig. 3. *DARS-AS1* functional associated with PACT. a, *DARS-AS1* is associated with cytoplasmic fractions. Total RNA of SW620 cells were separated into cytoplasmic (Cyto, GAPDH as marker) and nuclear (Nucl, LaminB and *MALAT1* as

markers) fractions. **b**, Proteins from RNA pull-down assay were validated by western blot using indicated antibodies. **c**, qPCR detected gene expression in cells overexpressed PACT or *DARS-AS1*. **d**, Immunoblot detected the knockdown efficiency of PACT in SW620. MTS assay measured the cell growth rate of PACT-knockdown or control (NC) SW620 cells. **e**, Colony formation of PACT-knockdown or control (NC) SW620 cells. **f-h**, Knockdown *DARS-AS1* induces cell apoptosis in HCT116 (**f**), HepG2 (**g**) or MBA-MD-231 (**h**) cells, as revealed by flow cytometry. The statistic results were obtained by three repetitions. Data shown are means ± SD in triplicates experiments. *$p \leq 0.05$, **$p \leq 0.01$, ***$p \leq 0.001$, ns, not significant, by two-tailed Student's *t test*.

Supplementary Figure 4

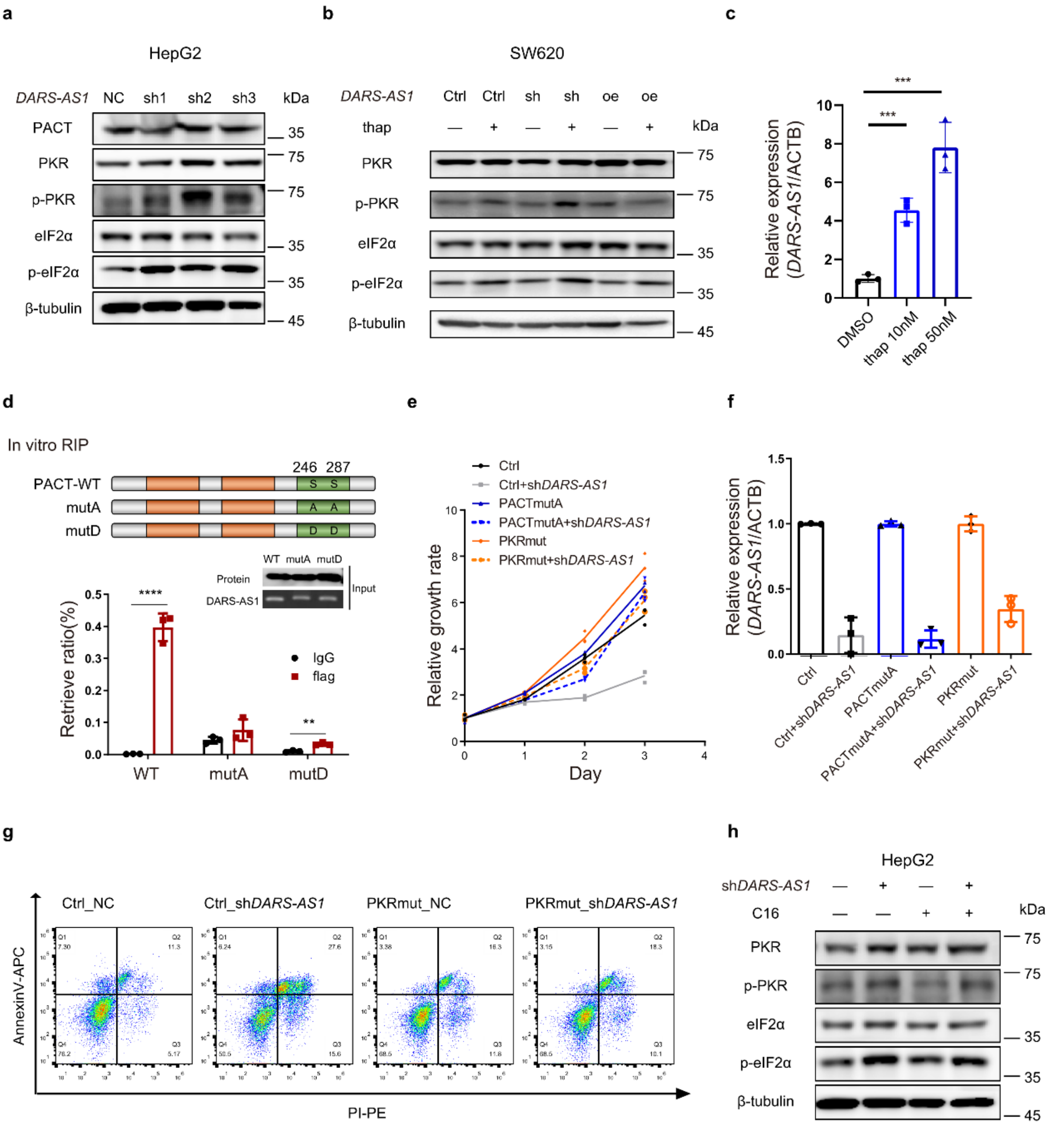

Supplementary Fig. 4. *DARS-AS1* suppresses PACT-mediated PKR activation. a, Immunoblot detected the expression of indicated proteins. HepG2 cells were transfected with control shRNAs or *DARS-AS1*-shRNAs, followed by serum starvation. **b**, The expression level of *DARS-AS1* alters cell sensitivity of thapsigargin. SW620 cells were transfected with *DARS-AS1* shRNAs, *DARS-AS1*-overexpression plasmids or control plasmids. Cells were treated by thapsigargin for 48 hours and immunoblot were performed using indicated antibodies. **c**, qPCR detected the expression of *DARS-AS1* in SW620 cell treated by thapsigargin or DMSO. **d**, In vitro RIP assays

were performed using purified wild type or mutant flag-tagged PACT. Retrieved RNA was validated by RT-qPCR. **e-f**, Growth rate of SW620-ctrl cells, cells overexpressing PKR mutant (PKRmut) or PACT mutant (PACTmutA) (**e**). Cells were transfected with control shRNAs or *DARS-AS1*-shRNAs. The knockdown efficiency of *DARS-AS1* were validated by qPCR (**f**). **g**, Inactive mutant PKR compensated SW620 cell apoptosis induced by *DARS-AS1*, as revealed by flow cytometry. **h**, Immunoblot using indicated antibodies were performed in HepG2 cells. Cells transfected with control shRNAs or *DARS-AS1*-shRNAs were treated by serum starvation, suppling with PKR inhibiter C16 or DMSO. Data shown are means ± SD in triplicates experiments. *p ≤ 0.05, **p ≤ 0.01, ***p ≤ 0.001, ****p ≤ 0.0001, by two-tailed Student's *t test*.

Supplementary Figure 5

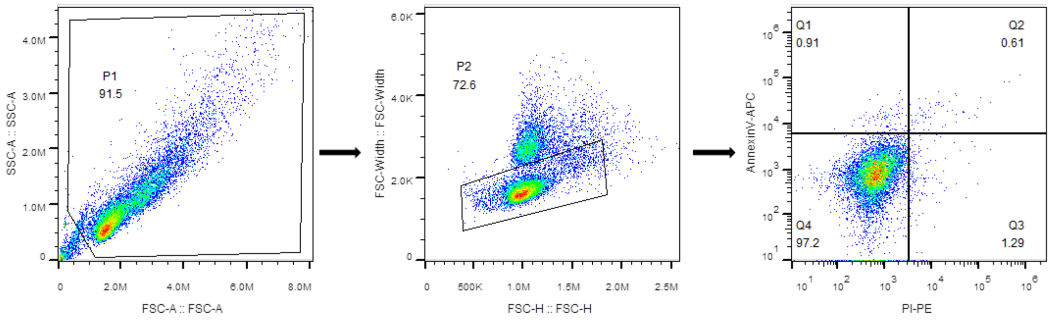

Supplementary Fig. 5. Example of the gating strategy for flow cytometry.

Supplementary Figure 6

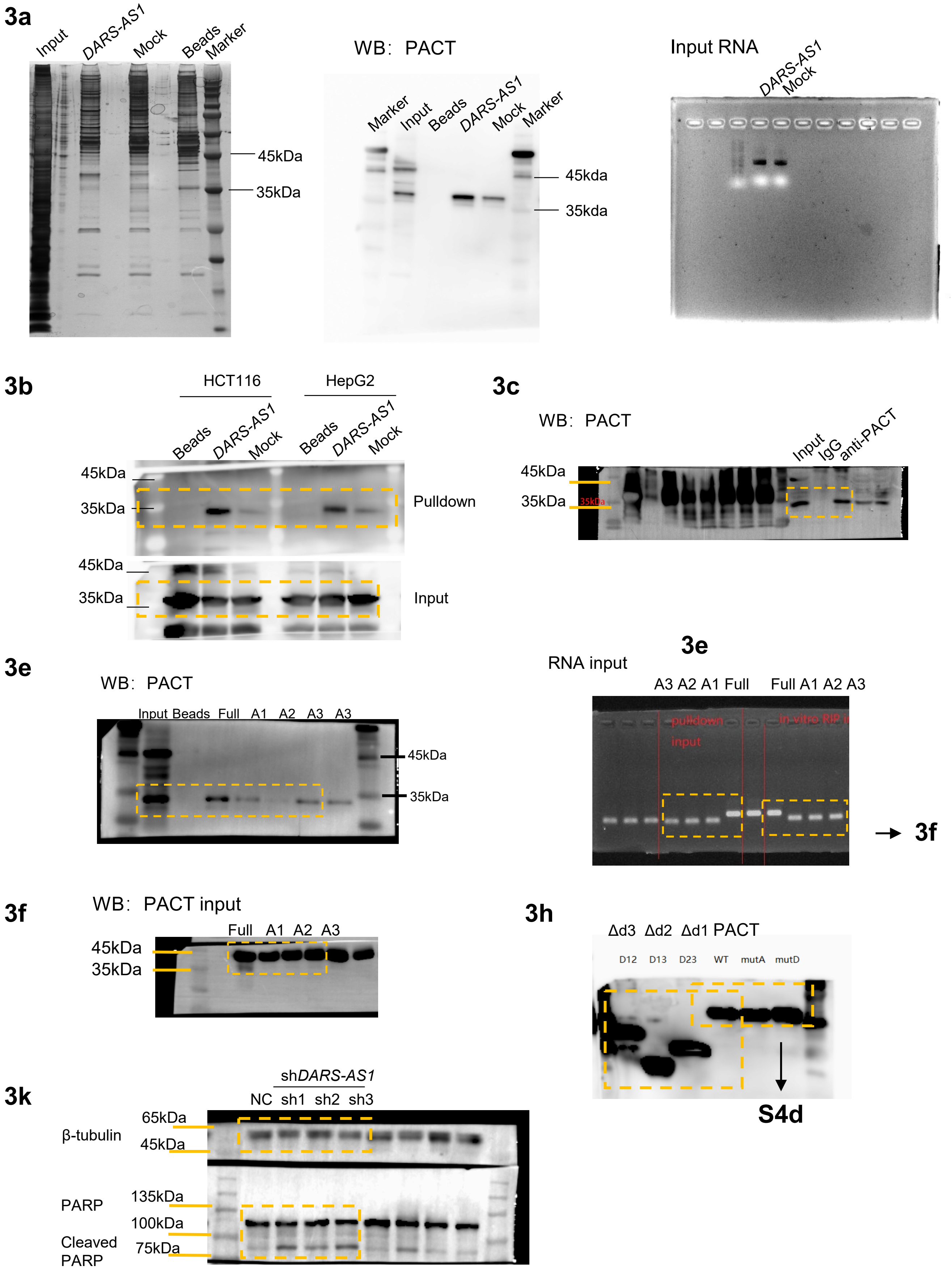

Supplementary Fig. 6 Unedited gel for Fig. 3

Supplementary Figure 7

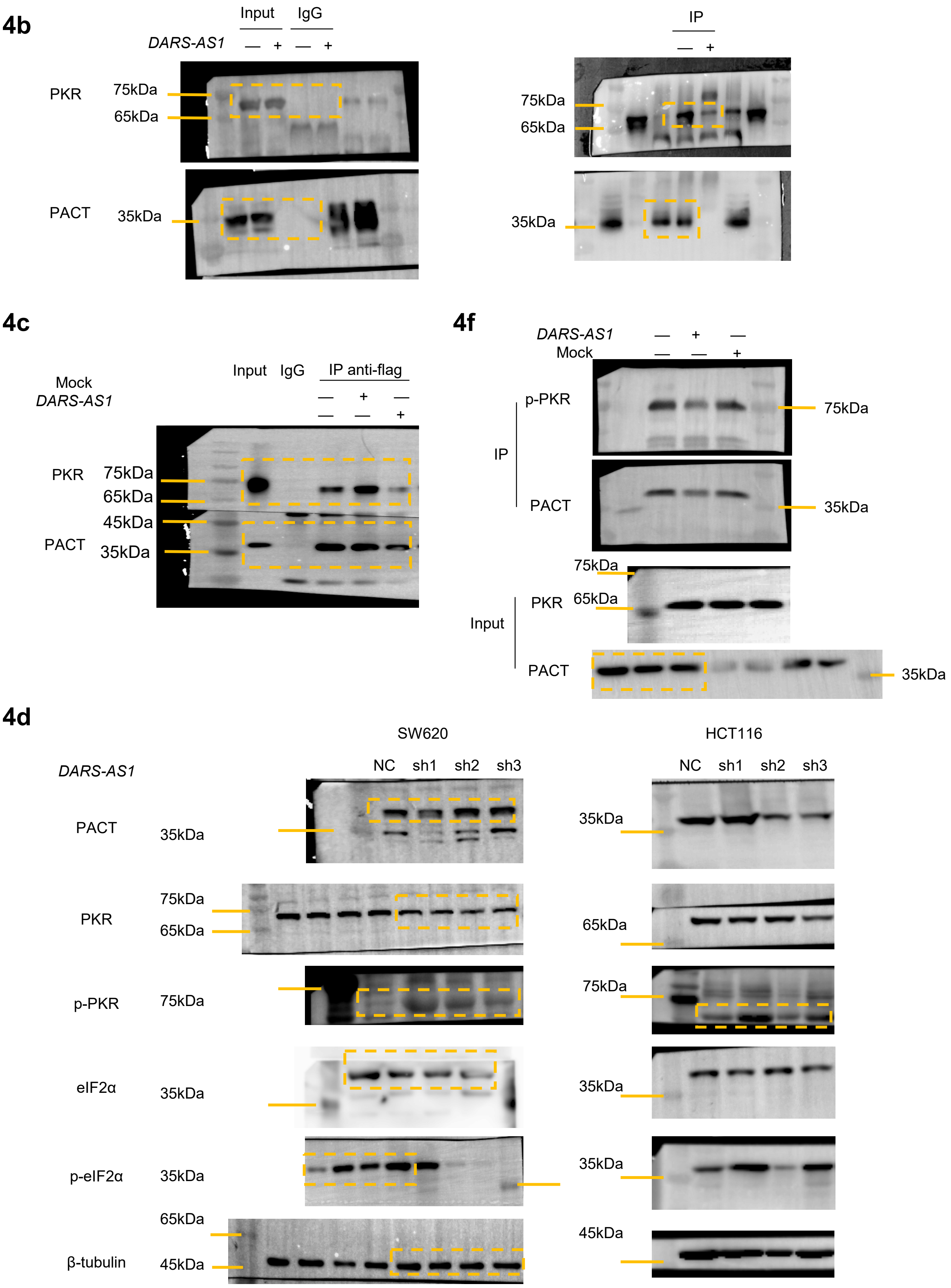

Supplementary Fig. 7 Unedited gel for Fig. 4b, 4c, 4d and 4f

Supplementary Figure 8

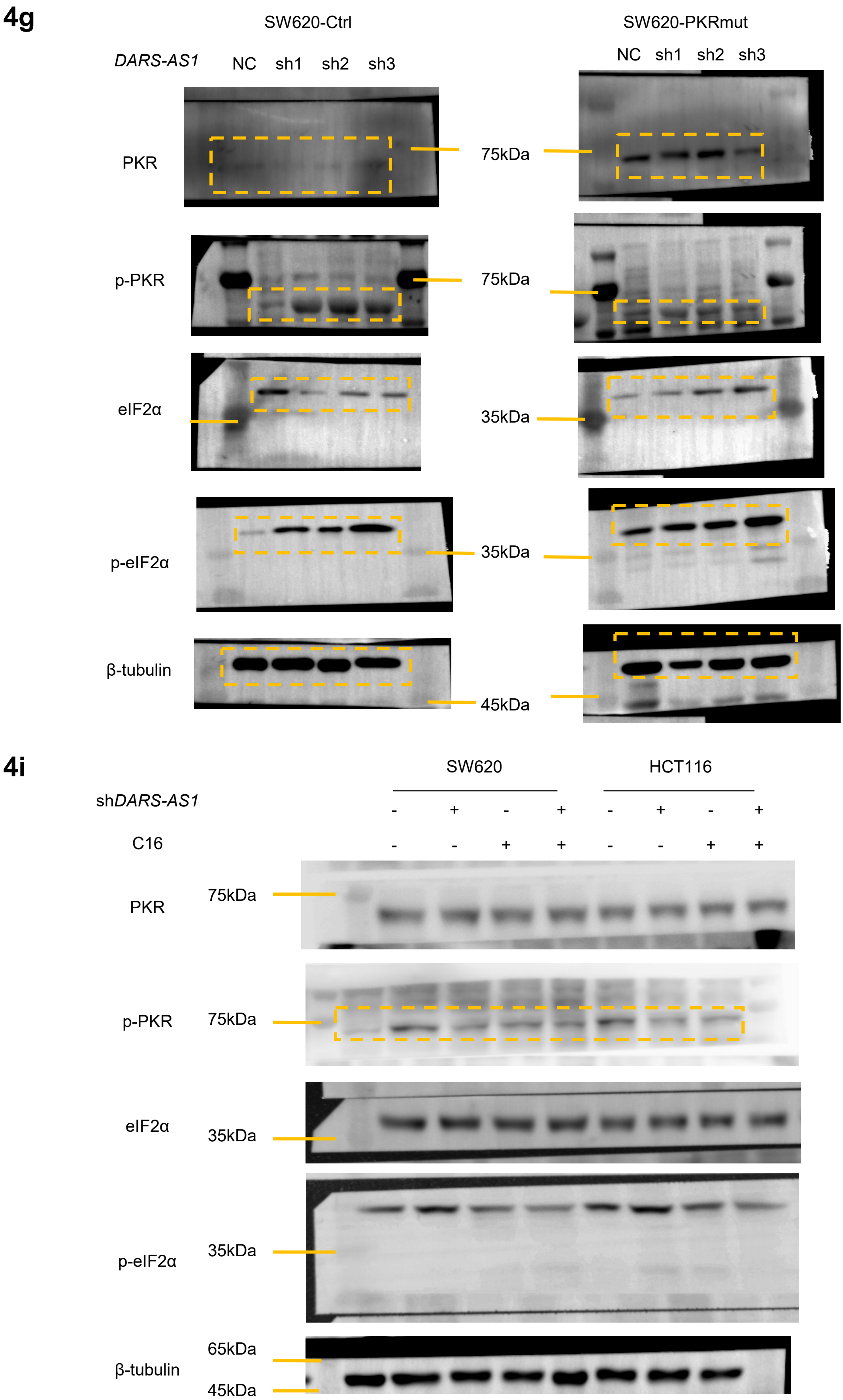

Supplementary Fig. 8 Unedited gel for Fig. 4g and 4i

Supplementary Figure 9

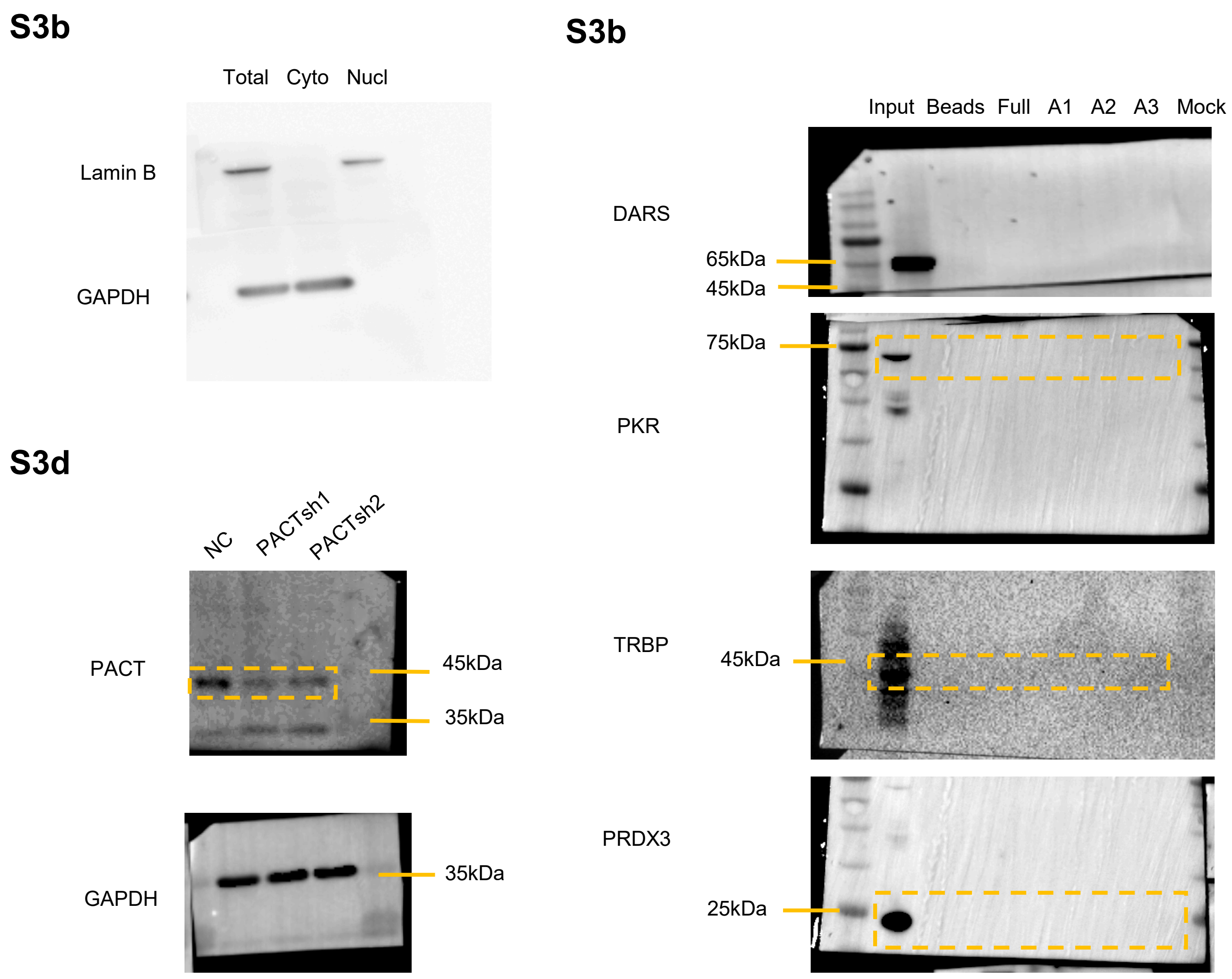

Supplementary Fig. 9 Unedited gel for Fig. S3

Supplementary Figure 10

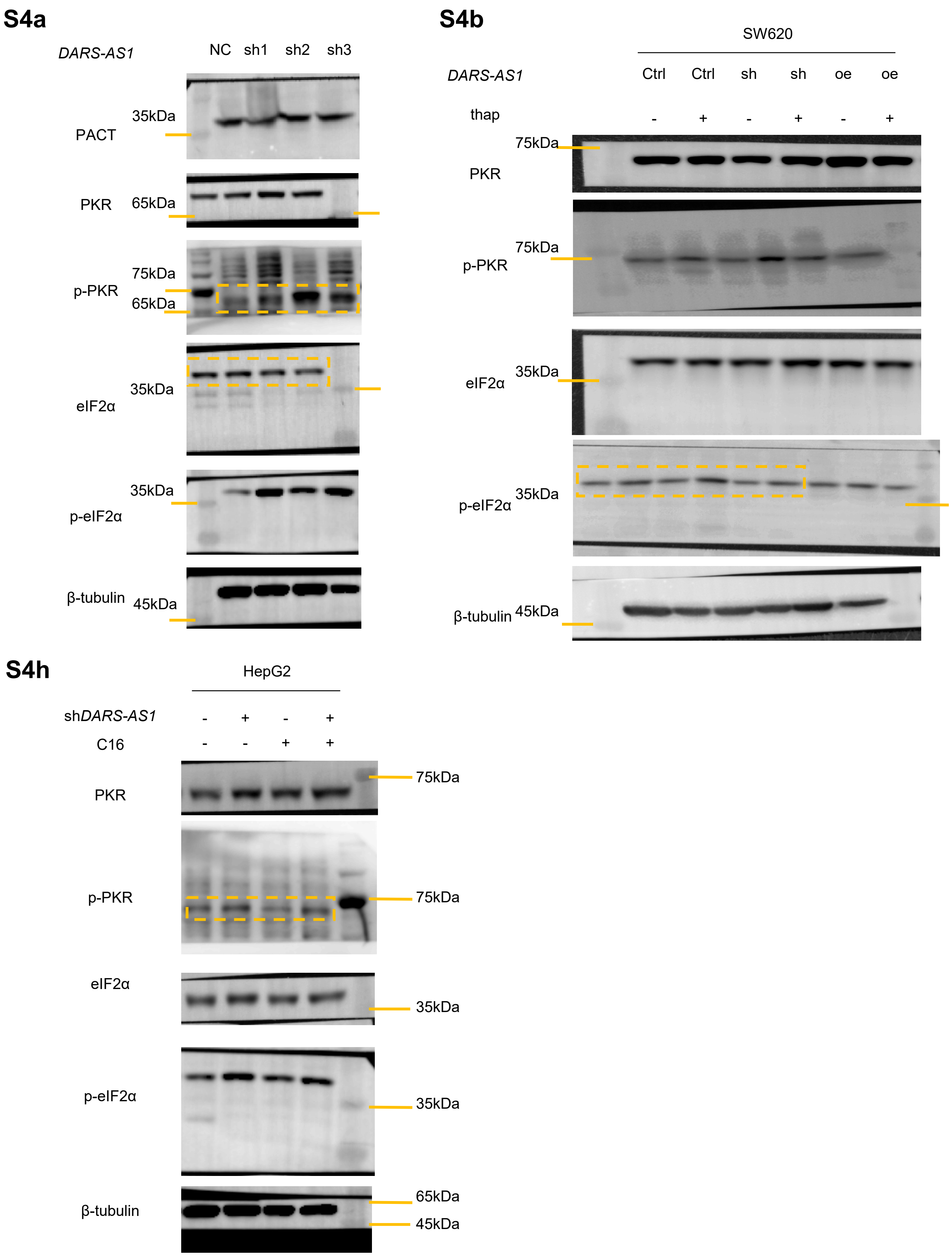

Supplementary Fig. 10 Unedited gel for Fig. S4

Supplementary Table 1. The summary of CRISPRi screening results

Sample	total reads	sequencing depth
SW620_day0_rep1	5124383	652
SW620_day0_rep2	4834548	615
SW620_day0_rep3	4298508	547
SW620_day0_rep4	4734102	603
SW620_day17_rep1	4783560	609
SW620_day17_rep2	4302854	548
SW620_day17_rep3	4134830	526
SW620_day17_rep4	4183347	533

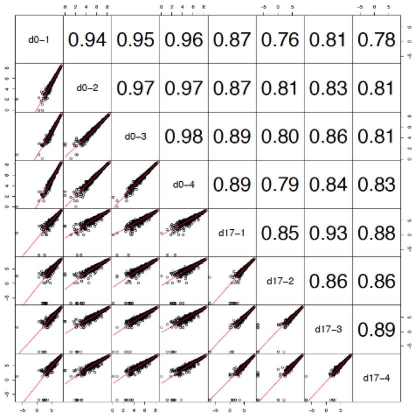

Left: The total reads and sequencing depth of screening.
Right: Replicate consistency (R-Squared) of each two samples.

Supplementary Table 2. Sequences of *DARS-AS1* shRNAs and sgRNAs

Name	Sequences（5 ' - 3 '）
shNC	CAACAAGATGAAGAGCACCAA
DARS-AS1 sh1	GGCTTATAAGGGACTATATCT
DARS-AS1 sh2	GAAGGACAAGCAACTTCAAAT
DARS-AS1 sh3	GAGCTTGTTTAGTGTGCAAAT
DARS-AS1 sg1	CCTACTAGAAGCGTGTCCCC
DARS-AS1 sg2	TGCCTTACGGAAGCCTGCGA

Supplementary Table 3. The C_t value of *DARS-AS1* in cell lines detected by RT-qPCR

C_t value	*ACTB*#			*DARS-AS1*			*DARS-AS1*(mean) - *ACTB*(mean)*
SW620	15.715	15.688	15.669	24.772	24.682	24.640	9.007
HCT116	15.236	14.573	14.421	23.809	23.810	23.767	9.052
HepG2	14.803	15.196	15.084	26.180	26.150	26.160	11.136
MDA-MB-231	15.700	15.780	15.750	26.800	26.580	26.690	10.947
A549	15.310	15.010	15.170	27.663	27.552	27.190	12.305

#: Using *ACTB* as internal control.

*: Mean: the average C_t of triplicates experiments.

Supplementary Table 4. Primers for in vitro transcription

Name	Sequences（5 ' - 3 '）
DARS-AS1-F	TAATACGACTCACTATAGGCGGTCCGGAGAGG
DARS-AS1-R	CCTGTGTTAGTTTGCTGAGAAT
1-384nt-F	TAATACGACTCACTATAGGCGGTCCGGAGAGG
1-384nt-R	CTTGGCTAAGAAAGACAGGATCC
192-576nt-F	TAATACGACTCACTATAGCGTCCATGATCTCCCGC
192-576nt-R	TGCTTGGAAATTTGAAGTTGC
384-768nt-F	TAATACGACTCACTATAGGACTGGTCTCTTTTCTCCAATG
384-768nt-R	CCTGTGTTAGTTTGCTGAGAATG
antisense-F	TAATACGACTCACTATAGCCTGTGTTAGTTTGCTGAGAAT
antisense-R	GCGGTCCGGAGAGG
mock-F	TAATACGACTCACTATAGATGGTGAGCAAGGGCG
mock-R	CTTGTACAGCTCGTCCATGC

Supplementary Table 5. Primers for RT-qPCR

Name	Sequences（5＇- 3＇)
DARS-AS1-F	CATCGGGACACGGAACTGG
DARS-AS1-R	TGCAAAGAACTGCAGAAGACAC
MALAT1-F	AGTACTGTTCTGATCCCGCTGCT
MALAT1-R	CTAAAGGCTTCAGTCCCCTCCCA
DARS-F	GCCTGAGGCAGAAGGAGAAGAG
DARS-R	ATGGCAGATGCCAGACTGGAGA
GAPDH-F	GTCTCCTCTGACTTCAACAGCG
GAPDH-R	ACCACCCTGTTGCTGTAGCCAA
PACT-F	CCCTTAATGCCTGACCCTTCCA
PACT-R	CAGGAAGTCTCCAGCCATGATG
PKR-F	GAAGTGGACCTCTACGCTTTGG
PKR-R	TGATGCCATCCCGTAGGTCTGT
ACTB-F	ATCTGGCACCACACCTTCTAC
ACTB-R	CAGCCAGGTCCAGACGCAGG
