# Supplementary material for: Long non-coding RNA DARS-AS1 promotes tumor progression by directly suppressing PACT-mediated cellular stress
Source: Commun Biol. 2022 Aug 15;5:822. doi: 10.1038/s42003-022-03778-y (PMC9378715; doi:10.1038/s42003-022-03778-y)
Supplement: Supplementary file 3 — Description of Additional Supplementary Files [file 42003_2022_3778_MOESM3_ESM.pdf]

Description of Additional Supplementary Files

File name: Supplementary Data 1
Description: Detailed information of CRISPRisgRNAs library.

File name: Supplementary Data 2
Description: The source data behind the graphs in the main manuscript.

File name: Supplementary Data 3
Description: The source data behind the graphs in Supplementary Files.
