# Supplementary material for: Long non-coding RNA DARS-AS1 promotes tumor progression by directly suppressing PACT-mediated cellular stress
Source: Commun Biol. 2022 Aug 15;5:822. doi: 10.1038/s42003-022-03778-y (PMC9378715; doi:10.1038/s42003-022-03778-y)
Supplement: Supplementary file 7 — Reporting Summary [file 42003_2022_3778_MOESM7_ESM.pdf]

nature research

Corresponding author(s): Dong Wang

Last updated by author(s): Jul 2, 2022

Reporting Summary

Nature Research wishes to improve the reproducibility of the work that we publish. This form provides structure for consistency and transparency in reporting. For further information on Nature Research policies, see our Editorial Policies and the Editorial Policy Checklist.

Statistics

For all statistical analyses, confirm that the following items are present in the figure legend, table legend, main text, or Methods section.

n/a	Confirmed	
☐	☒	The exact sample size (*n*) for each experimental group/condition, given as a discrete number and unit of measurement
☐	☒	A statement on whether measurements were taken from distinct samples or whether the same sample was measured repeatedly
☐	☒	The statistical test(s) used AND whether they are one- or two-sided<br>*Only common tests should be described solely by name; describe more complex techniques in the Methods section.*
☒	☐	A description of all covariates tested
☒	☐	A description of any assumptions or corrections, such as tests of normality and adjustment for multiple comparisons
☐	☒	A full description of the statistical parameters including central tendency (e.g. means) or other basic estimates (e.g. regression coefficient) AND variation (e.g. standard deviation) or associated estimates of uncertainty (e.g. confidence intervals)
☐	☒	For null hypothesis testing, the test statistic (e.g. *F*, *t*, *r*) with confidence intervals, effect sizes, degrees of freedom and *P* value noted<br>*Give P values as exact values whenever suitable.*
☒	☐	For Bayesian analysis, information on the choice of priors and Markov chain Monte Carlo settings
☒	☐	For hierarchical and complex designs, identification of the appropriate level for tests and full reporting of outcomes
☐	☒	Estimates of effect sizes (e.g. Cohen's *d*, Pearson's *r*), indicating how they were calculated

Our web collection on statistics for biologists contains articles on many of the points above.

Software and code

Policy information about availability of computer code

Data collection	The FPKM values of DARS-AS1 in TCGA normal and tumor samples and the clinical information of the patients were downloaded from UCSC Xena (http://xena.ucsc.edu).
Data analysis	GraphPad Prism (Version 8) were used for data analysis and graphing; ImageJ 1.47v was used for imaging analysis; R (Version 3.5.1) and Rstudio (Version 1.0.136) were used for Kaplan-Meier analysis; Perl (Version 5.18) was used to merge different files.

For manuscripts utilizing custom algorithms or software that are central to the research but not yet described in published literature, software must be made available to editors and reviewers. We strongly encourage code deposition in a community repository (e.g. GitHub). See the Nature Research guidelines for submitting code & software for further information.

Data

Policy information about availability of data

All manuscripts must include a data availability statement. This statement should provide the following information, where applicable:

- Accession codes, unique identifiers, or web links for publicly available datasets
- A list of figures that have associated raw data
- A description of any restrictions on data availability

The main data supporting the findings of this study are available within the manuscript and its Supplementary Information files. All other data are available from the corresponding author upon reasonable request. Correspondence and requests for materials should be addressed to Dong Wang (dwang@cdutcm.edu.cn)

Field-specific reporting

Please select the one below that is the best fit for your research. If you are not sure, read the appropriate sections before making your selection.

☒ Life sciences ☐ Behavioural & social sciences ☐ Ecological, evolutionary & environmental sciences

For a reference copy of the document with all sections, see nature.com/documents/nr-reporting-summary-flat.pdf

Life sciences study design

All studies must disclose on these points even when the disclosure is negative.

Sample size	Sample size for in vitro and in vivo experiments was chosen based on previous studies and experimental knowledge. The sample size was clearly stated in the figure legend or in the method section.
Data exclusions	No data was excluded.
Replication	For the biochemical assay, each experiment was replicated for 3 times and the results were reproduced each time.
Randomization	Animals were randomly allocated into different groups.
Blinding	Human research participants were not involved in this study. No blinding used in this study. Whenever possible, the data analysis was performed in an unbiased way.

Reporting for specific materials, systems and methods

We require information from authors about some types of materials, experimental systems and methods used in many studies. Here, indicate whether each material, system or method listed is relevant to your study. If you are not sure if a list item applies to your research, read the appropriate section before selecting a response.

Materials & experimental systems

n/a	Involved in the study
☐	☒ Antibodies
☐	☒ Eukaryotic cell lines
☒	☐ Palaeontology and archaeology
☐	☒ Animals and other organisms
☒	☐ Human research participants
☒	☐ Clinical data
☒	☐ Dual use research of concern

Methods

n/a	Involved in the study
☒	☐ ChIP-seq
☐	☒ Flow cytometry
☒	☐ MRI-based neuroimaging

Antibodies

Antibodies used	anti-PACT, Abcam (ab31967); anti-PKR, Abcam (ab184257); anti-PKR (phospho T451), Abcam (ab81303); anti-flag, Abcam (ab125243); anti-eIF2α, Abclonal (A0764); anti-eIF2α (phospho S51), Abcam (ab32157); anti-PACT (phosphor S246), Abgent (AP7744b); anti-βtubulin, CST (2128); Normal mouse IgG, CST (5415S); Normal rabbit IgG, CST (2729S).
Validation	The antibodies used in this study were bought from the commercial companies. The validation statement for each antibody was provided in the manufacture's website.

Eukaryotic cell lines

Policy information about cell lines

Cell line source(s)	All cell lines were purchased from National Infrastructure of Cell Line Resource, Beijing, China.
Authentication	The authentication of the cell lines was confirmed by National Infrastructure of Cell Line Resource.
Mycoplasma contamination	All cell lines have been tested free for mycoplasma contamination by PCR.
Commonly misidentified lines (See ICLAC register)	No commonly misidentified cell lines were used.

Animals and other organisms

Policy information about studies involving animals; ARRIVE guidelines recommended for reporting animal research

Laboratory animals	Nude mice were used in this study. All the nude mice were BALB/c background, female, and 6-8 week old.
Wild animals	Wild animals were not involved in this study.
Field-collected samples	Field-collected samples were not involved in this study.
Ethics oversight	The studies on animals were performed under relative guidelines and were approved by the Animal Care and Use Committee of Tsinghua University.

Note that full information on the approval of the study protocol must also be provided in the manuscript.

Flow Cytometry

Plots

Confirm that:

☒ The axis labels state the marker and fluorochrome used (e.g. CD4-FITC).

☒ The axis scales are clearly visible. Include numbers along axes only for bottom left plot of group (a 'group' is an analysis of identical markers).

☒ All plots are contour plots with outliers or pseudocolor plots.

☒ A numerical value for number of cells or percentage (with statistics) is provided.

Methodology

Sample preparation	Cells were digested and washed twice with PBS, then staining with Annexin V Apoptosis Detection Kit (eBioscience 88-8007-74) following the instructions. Briefly, cells were resuspended in 1X binding buffer containing APC-conjugated Annexin V and incubated in dark for 10-15 minutes at room temperature. After washing with 1X binding buffer, cells were resuspended in 1X binding buffer containing Propidium Iodide. Cells were filtered with 70um filter and analyzed by flow cytometry.
Instrument	LSRFortessa (BD Bioscience)
Software	All samples were analyzed with LSRFortessa (BD Bioscience) and data were analyzed using FlowJo software.
Cell population abundance	At lest 10,000 cells (about more than 70% of total) are analyzed in the final gate of each sample.
Gating strategy	Flow cytometry was used to analyze apoptosis. FSC/SSC gates the entire cell population, excluding obvious debris. FSC-H/FSC-W gates single-cell population. The last cytograms shown Annexin V-APC vs. PI-PE.

☒ Tick this box to confirm that a figure exemplifying the gating strategy is provided in the Supplementary Information.
